# Family study of a novel mutation of mucopolysaccharidosis type VI with a severe phenotype and good response to enzymatic replacement therapy: Case report

**DOI:** 10.1097/MD.0000000000012872

**Published:** 2018-10-19

**Authors:** Myriam Ley-Martos, Juan M. Guerrero, Marta Lucas-Javato, Cristina Remón-García, J. Raúl García-Lozano, Cristóbal Colón, Pablo Crujeiras, Daniel Rodrigues, Pedro Paúl-Sánchez, Hada C. Macher

**Affiliations:** a Pediatric Neurology and Rare Diseases Unit, Department of Pediatry, Hospital Universitario Puerta del Mar, Cádiz; b Molecular Diagnosis and Rare Diseases Laboratory, Department of Clinical Chemistry, Hospital Universitario Virgen del Rocío and Instituto de Biomedicina de Sevilla (IBiS), Sevilla; c Pneumology Unit, Department of Pediatry, Hospital Universitario Puerta del Mar, Cádiz; d Molecular Diagnosis Unit, Department of Clinical Immunology, Hospital Universitario Virgen del Rocío and Instituto de Biomedicina de Sevilla (IBiS), Sevilla; e Diagnosis and Treatment of Congenital Metabolic Diseases Unit, Department of Pediatry, Hospital Clínico Universitario and Instituto de Investigacion en Salud (IDIS), Santiago de Compostela; f Department of Pediatry, Hospital Universitario, Ceuta, Spain.

**Keywords:** arylsulfatase B, lysosomal disorder, mucopolysaccharidosis type V, rare diseases

## Abstract

**Rationale:**

Mucopolysaccharidosis type VI (MPS VI) or Maroteaux-Lamy syndrome is produced by the deficiency of the enzyme arylsulfatase B, responsible for the hydrolysis of N-acetyl-D-galactosamine, chondroitin sulfate, and dermatan sulfate.

**Patient concerns:**

A 3-year-old male with Moroccan origins is the index case. He had healthy consanguineous parents and 4 healthy brothers and sisters. The patient showed a wide spectrum of symptoms including skeletal dysplasia and short stature with elevated glycosaminoglycans (GAGs) in urine.

**Diagnoses, interventions, and outcomes:**

GAGs were quantified by spectrometry method with 1,9-dimethylen blue in 24-hour urine samples. The qualitative analysis of urine GAGs was obtained by thin-layer chromatography to determine the predominant presence of dermatan sulfate. The activities of both arylsulfatase B and beta-galactosidase as well as genetic studies were performed in dried blood spots. The genetic study was performed with deoxyribonucleic acid by massive sequencing a of lisosomal storage diseases. Results showed a new mutation c.263A > C with the severe phenotype in homozygous in the patient. The familiar study of *ARSB* and *GLB1* genes presented some asymptomatic SNPs but with a discrete decrease in the activity of arylsulfatase B and beta-galactosidase. After an early detection by pediatricians, and both enzymatic and genetic confirmation, the patient had a good response to substitutive enzymatic treatment with galsulfase.

**Lessons:**

Mucoplysaccharidosis type VI is an autosomal recessive rare disease characterized by a lysosomal storage disorder. Although a number of mutations have been already associated to the disease, we have found a new mutation located in the arylsulfatase B enzyme gene. We have described that this mutation is the ultimate cause of a severe presentation of the disease.

## 1 Introduction

Mucopolysaccharidosis type VI (MPS VI) or Maroteaux-Lamy syndrome is an autosomal recessive lysosomal storage disorder described by Pierre Maroteaux and Maurice Lamy in 1963^[1]^ and due to mutations in the arylsulfatase B gene (*ARSB*) located in chromosome 5 (5q13–5q14).^[2]^ Pathogenic mutations of this gene result in either reduced or absence of the enzyme arylsulfatase B (ASB) activity, also called N-acetylgalactosamine 4-sulfatase (E.C.3.1.6.12), leading to incomplete degradation and cellular accumulation of the glycosaminoglycan (s) (GAGs). The epidemiological studies of MPS VI are estimated in a range from 1 in 455,000 newborns.

The clinical presentation of MPS VI varies greatly with respect to age at onset and rate of disease progression. Although descriptive classification systems have mostly described patients as rapidly progressing (with severe symptoms) or slowly progressing (with mild or attenuated symptoms), an intermediate stage has also been described. The onset of the rapidly progressing form is, in most cases, before 2 or 3 years of age, showing impaired mobility by 10 years of age, absence or delayed puberty, cervical spinal cord compression, respiratory insufficiency, and surgical complications. Patients with the rapidly progressing form were frequently reported as dying from heart failure in the second or third decade of life. Growth often slows down after the first year of life, with complete cessation at 3 to 4 years of age.^[3,4]^

Other physical findings may include thoracic deformity (pectus carinatum), stiff and contracted joints, scoliosis or kyphosis (gibbus malformation), macrocephaly, hepatosplenomegaly, protruding abdomen, umbilical and/or inguinal hernia, coarse facial features including frontal bossing, a depressed nasal bridge, enlarged tongue, gingival hypertrophy, delayed dental eruption, and hypertrichosis.^[5]^ Patients may have labored breathing, loud snoring with sleep apnea, thick nasal discharge, frequent sinusitis, or otitis media arising from narrowed airways and thick mucous secretions.^[6]^

Loss of hearing, involving both conductive and neurosensory mechanisms, is common and may lead to the impression that the patient may have developmental delay. Vision is often compromised by slowly increasing corneal clouding, but can also show rapid deterioration related to optic nerve damage from increased intracranial pressure or compression along the optic nerve.^[7]^

The radiological findings that are characteristic of MPS VI are described as “dysostosis multiplex.” Typical radiological findings include thickened, short metacarpal bones with proximal pointing and thin cortices, carpal bones that are irregular and hypoplastic, and tarsal bones that have irregular contours, a dysplastic femoral head, severe hip dysplasia, abnormal development of vertebral bodies of the spine, paddle-shaped widened ribs and short, thick irregular clavicles, hypoplastic distal ulna and radius, thickened diploic space, and abnormally shaped J-shaped sella in the cranium. Slowly progressing MPS VI patients may not demonstrate all the above characteristics of dysostosis multiplex.

Other MPS also exhibit a slow growth pattern. This is the case of MPS IVB or Morquio B syndrome, in which there is a deficiency of beta-galactosidase-1, a lysosomal hydrolase that cleaves the terminal beta-galactose from ganglioside substrates and other glycoconjugates, and encoded by gen *GLB1*.^[8]^ MPS IVB is an autosomal recessive disorder characterized by skeletal dysplasia and corneal clouding. The growth is reduced and there is no central nervous system involvement, and intelligence is normal with increased urinary keratan sulfate excretion.^[9]^ But there may also be a nonkeratansulfate-excreting form of Morquio syndrome, the so-called type C.

Nowadays, an enzyme replacement therapy for MPS VI with galsulfase (recombinant human [rh] ASB; Naglazyme, BioMarin Pharmaceutical Inc., Novato, CA) exists. It is shown to be effective in improving endurance and pulmonary function, reducing intracellular GAG accumulation, and stabilizing cardiac manifestations. Thus, the effect of galsulfase replacement therapy on the growth of MPS VI patients depends on age of treatment initiation and pretreatment levels of urine GAG.^[10,11]^

## 2 Case report

### 2.1 Clinical characteristics

A 3-year-old male with Moroccan origins is the index case. His main query reason to consult was a disharmonic low size. He has healthy consanguineous parents and 4 healthy brothers and sisters. All of them were informed and consent was given for a familial enzymatic and genetic study for lisosomal storage diseases. The index case sent 24 hours urine, dried blood spot (DBS), and ethylene diamine tetraacetic acid (EDTA) blood. His father sent DBS sample and EDTA blood, but his mother, 2 sisters, and 2 brothers live in Morocco and the only sample sent was DBS.

Urine quantitative analysis is based on the spectrometric determination of the binding of glycosaminoglycans (GAGs) with 1,9-dimethylen blue, in 24-hour urine samples. The absorbance readings are performed at 630 nm, and the reference values depend on age.

This method allows us to detect the excretion of GAGs increased in urine, but it is not possible to differentiate the type of GAGs excreted. The qualitative analysis of urine GAGs was obtained by thin-layer chromatography to determine the predominant presence of dermatan sulfate.

In the enzymatic analysis, the action of the beta-galactosidase enzyme present in the DBS sample is determined on the fluorometric substrate 4-methylumbelliferyl-beta-D-galactopyranoside, releasing 4-methylumbelliferyl, which, at alkaline pH, produces fluorescence, proportional to the enzymatic activity. We adapted the methods of Hein et al,^[12]^ and Ho and O’Brien^[13]^ to evaluate the enzymatic activity of arylsulfatase B (ARSB, EC 3.1.6.1) and beta-galactosidase (GLB, EC 3.2.1.23), respectively. For the ARSB, measured in DBS, a 3.2-mm punch was incubated 20 hours with 50 μL substrate 4-methylumbelliferyl-sulfate, following a 20-minute preincubation with 30 μL water and 20 μL inhibitor (lead acetate). Reaction was stopped with 300 μL of ethylenediamine. Stopping buffer was added to the blanks before the substrate.

Leukocytes were separated from EDTA blood using the Wizard Genomic deoxyribonucleic acid (DNA) Purification Kit (Promega, Madison, WI), and stored at −20°C until used. The leukocyte samples were diluted in 0.9% NaCl and were sonicated in an Ultrasonic Sonicator Processor BandelinSonopuls HD 2070. The Bradford method was used for measuring protein in leukocytes. Fluorescence (excitation 355 nm; emission 460 nm) was measured on a BMG Labtech spectrofluorometer, model Fluo Star Optima. Readings were corrected for blanks, and compared with 4-methylumbelliferone calibrators. Enzyme activities were expressed in micromoles of 4-MU product formed per hour/liter of blood (DBS samples) or nanomoles per hour/mg of protein (leukocytes).

For DNA extraction from DBS samples, 6 punch of 3.2 mm of every sample were preincubated with Casework Extraction Kit (Ref:DC6745 Promega) according to DNA IQ System-Small Sample Casework Protocol #TB296 and they were automatically extracted with MagNa Pure Compact instrument (Roche Diagnostics, Manheim, Germany) with the Magna Pure Compact Nucleic Acid Isolation Kit I, according to the Total NA Plasma 100 400 V3 1 extraction protocol. Final elution of DNA was in 50 μL elution buffer and stored at −20°C until further use.

The genetic study was performed with DNA by massive sequencing of lisosomal storage diseases. Amplification was performed by multiplex PCR for coding regions and splicing sites of 81 genes with 15192 amplicons covering the 99.75% regions in a custom design Kit for Ion AmpliSeq. The sequencing was performed in an S5 Ion Torrent Platform. The *ARSB* and *GLB1* genes coding regions were 100% covered in the index case. However, DBS DNA samples do not amplify exon 2 of the GLB1 gene, but the index case, since DNA is extracted from EDTA blood samples, does have 100% coverage of both genes and the exon 2 of GLB1 is normal.

The index case was 1 of 5 children in a healthy consanguineous family, born at term with delivery by vacuum extraction. A set of anthropometric parameters were monitored at birth such as weight (3.130 g, percentile 25, −0.68 SD), length (50.5 cm, percentile 52, 0.07 SD), cephalic perimeter (35 cm, percentile 50, −0.01 SD), and Apgar score (9/10). He had normal growth and development in the first 2 years.

During the subsequent months, the clinical manifestations became progressively severe without psychomotor retardation. At the age of 3, he had stopped growing with body height of 84.4 cm (below first percentile 1, −2.92 SD), weight of 13 kg (below 11th percentile, −1.23 SD), body mass index of 16.64 (62nd percentile, 0.33 SD), cephalic perimeter of 51 cm (60th percentile, 0.27 SD), and sitting/carving size ratio of 0.563 (40th percentile, −0.26 SD). Musculo-skeletal deformities increased with hypertelorism, flattened nasal root, macrocephaly, bell-shaped thorax, dorsal kyphosis, genu valgus, metaphyseal widening, and short and broad fingers with slight stiffness of distal interphalange. He became a mouth breather with chronic nasal snoring with sporadic breathing pauses; an obstructive sleep apnea syndrome was confirmed by nocturnal respiratory polydraphy.

Otorhinolaryngologic evaluation showed hypertrophic tonsils, macroglossia, and abundant rhinorrhea without otitis. Echocardiogram showed a slightly dysplastic mitral and tricuspid valves, normo-functioning dysplastic aortic and pulmonary valves, and signs of interventricular septum hypertrophy (8.5 mm in diastole, Z score 2.6) with normal coronary pattern. He also had progressive hepatomegaly; his liver was 5 cm below the costal margin. His intelligence and other aspects of neurodevelopment were normal.

Urine GAGs were elevated (74 mg/mmol creatinine, normal range for his age <14.1). Qualitative analysis of urine showed the presence of dermatan sulfate with the absence of keratan sulfate. Peripheral blood leukocyte ASB activity levels were completely abolished (0 nmol/h/mg, normal range 5.4–63.0). The ASB activity was also studied in dried blood spot (DBS) samples in the index case, confirming his ASB reduced activity, while both parents, his brothers and sisters, showed values at the lower end of the reference interval, compatible with the carrier state (Table 1). Beta-galactosidase enzyme activity levels were found to be reduced in all the family members, although there was no clinical evidence of Morquio B syndrome (Table 1).

**Table 1:**
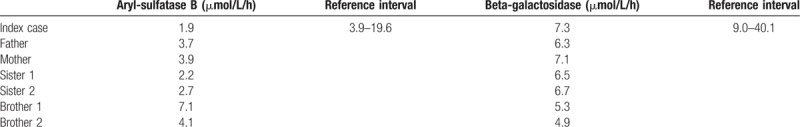
Aryl-sulfatase B and beta-galactosidase activities in dried blood spots samples of the family.

**Figure 1:**
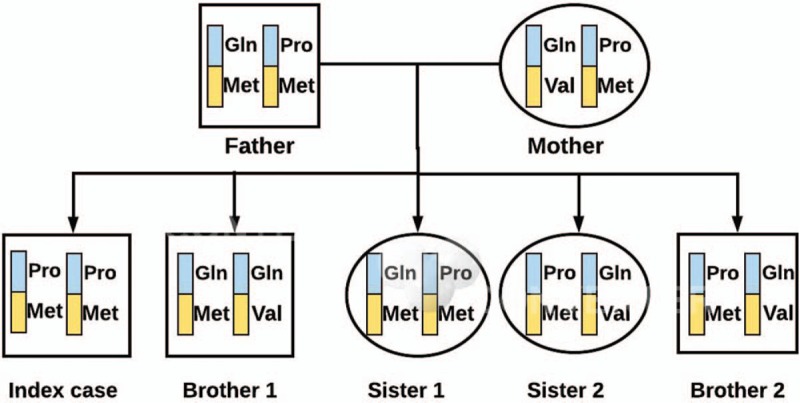
Hereditary pattern of MPS VI in the familial study. Blue and yellow bands represent exons 1 and 5, respectively. MPS VI = mucopolysaccaharidoses type VI.

Molecular characterization of the *ARSB* gene was performed for identification of all the possible deleterious and potential disease causing mutations. A single-nucleotide polymorphism (SNP) mutation was detected in homozygosis in the *ARSB* gene (NM_000046) at position Chr5:78280809 (c.263A > C) leading to a protein change p.Gln88Pro. Analysis of the variant was performed with Polyphen2 from, which it was described as a disease causing variant existing in homozygous state at the first exon of *ARSB* gene. This variant was not described in either Pubmed or the public access Human Genome Mutations Database (HGMD) (Table 2). The patient has another SNP variant in heterozygosis at position Chr5:78181477, in exon5 (c.1072 G > A), producing a change p.Val358Met described as benign in ClinVar (rs1065757) and as pathological in public access HGMD (Table 2). The hereditary pattern of MPS6 in this family is shown in Fig. 1.

**Table 2:**
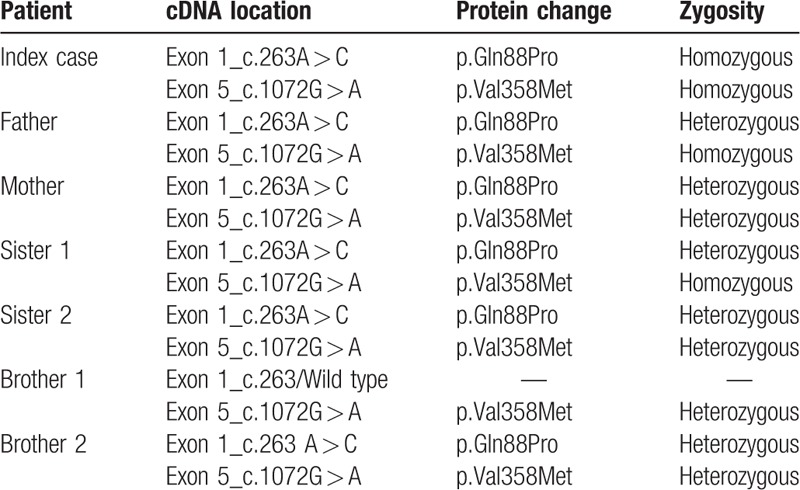
Punctual mutations found in *ARSB* gene.

Molecular characterization of the GL*B1* gene (NM_000404.3) was also performed for identification of all the possible deleterious and potential diseases causing mutations (Table 3). Two SNPs were detected in exon 1 of *GLB1* gene (NM_000404.3). The first variant existing in homozygous state in all family was at position Chr3:33138544 (c.34T > C) without protein change (p.Leu12 = ), classified by ClinVar as benign/likely benign (rs7614776). The second variant existing was at position Chr3:33138549 (c.29C > T) leading to a protein change (p.Prp10Leu). This variant was homozygous in the mother, wild type in the father, and in heterozygous state in all children, and described by ClinVar as benign/likely benign (rs7637099). All the family had a discrete reduction in the beta-galactosidase enzyme activity with no MPS IVB symptoms (Table 1).

**Table 3:**
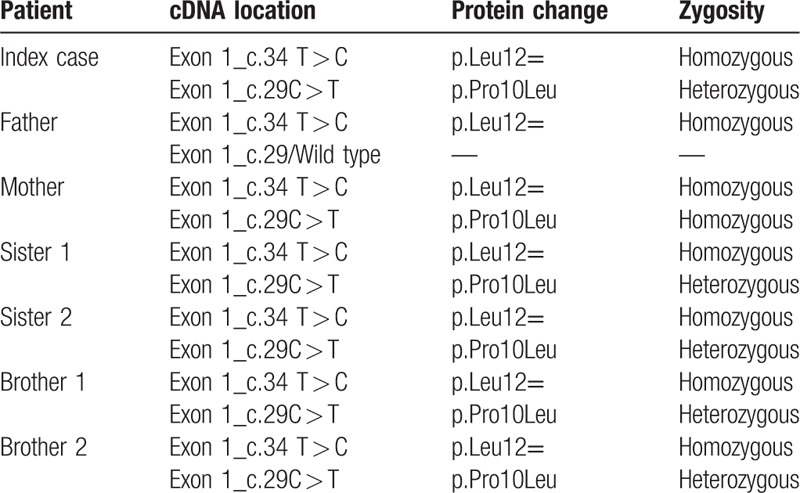
Punctual mutations found in *GLB1* gene.

None of the family members had symptoms of MPS VI, despite a discrete reduction in ASB activity (Table 1). They all had heterozygous state for the c.263A > C novel mutation. However, the index case, showing a drastic reduction in ASB activity, presented a homozygous state in the novel mutation. Brother 1 showed a wild-type pattern in c.236A with a normal ASB activity. The second variant (c.1072G > A) was homozygous in the index case, father, and sister 1, and heterozygous in the rest of family members (Table 2).

The patient was treated with galsulfase presenting a good tolerance and good clinical response. Within 1 year of treatment, he showed improvement in weight (from 13 to 15 kg; from percentile 11 to percentile 16), height (from 88.4 to 92.5 cm; still in percentile <1), and respiratory symptoms in both wakefulness and sleep. Medication for pulmonary hypertension had been withdrawn, and tonsillectomy and adenoidectomy were done with fibroscope in intensive care due to difficulty in intubation. In the last cardiology evaluation, the patient improved his interventricular septum hypertrophy (from 8.5 to 7.0 mm).

### 2.2 Informed consent

The patient's parents consented to our publishing this case report.

## 3 Discussion

The molecular analysis of the MPS VI patient and his family identified a novel mutation, a SNP variant. The p.Val358Met variant shows a possible comorbidity when it is homozygous in conjunction with the pathological mutation. But the novel homozygous mutation p.Gln88Pro is the cause of the disease since the patient has undetectable ASB activity in lymphocytes.

As a rare lysosomal storage disorder, there are only limited data available of MPS VI. However, it has been clearly described that the early initiation of galsulfase treatment is important to maximize growth potential in these patients.^[11]^ Particularly, sibling studies, in which 1 sibling begins therapy at a much younger age than the other, provides compelling evidence of benefits of early galsulfase treatment initiation.^[14,15]^ Whether the greater weight gain also results in reduced morbidity and increased survival remains to be studied.

Typically, patients with rapidly progressing disease experience growth delay at around 2 to 3 years of age. Although the mechanism of growth failure in patients experiencing a rapid disease progression is poorly understood, the accumulation of GAGs in growth plates and joints that leads to chondrocyte dysfunction, growth plate disorganization, and cytokine and inflammatory responses may contribute. It has been described that the effect of galsulfase treatment on height was more substantial in patients who started treatment at 6 years of age. The prevention of GAG accumulation in the growth plates may be a potential mechanism for improving growth in rapidly progressing patients.^[11]^

In our case, the patient started treatment with gaslsulfase at the age of 3 years showing a good tolerance, and also good and fast clinical response. He showed an improvement in weight (from 13 to 15 kg; from percentile 11 to percentile 16), height (from 88.4 to 92.5 cm; percentile is still <1), and respiratory symptoms, both in wakefulness and in sleep in a year. Medication for pulmonary hypertension was withdrawn because it was not necessary by that time.

Another common clinical manifestation in MPS VI is cardiac involvement, which is a major cause of mortality. Particularly, the ventricular hypertrophy and functional abnormalities of the mitral and aortic valves are progressive in individuals who are not treated,^[16]^ probably aggravated by pulmonary hypertension. In the last cardiology evaluation our patient improved his interventricular septum hypertrophy with respect to the previous echocardiography performed 1 year before (from 8.5 to 7 mm in diastole, Z score from 2.6 to1.8).

Regarding the slow improvement in height, the reduced activity of beta-galactosidase is not enough to express symptoms of MPS IVB illness, but it could be a possible cause of less response in growth to the substitutive enzymatic treatment for MPS VI. Age-dependent impacts of enzymatic replacement therapy are well described in literature.

## 4 Conclusions

An early diagnosis and prompt initiation of galsulfase treatment seem to be of critical importance.^[17,18]^ However, MPS VI diagnosis is not included in newborn screening programs in most countries. This is probably due to the fac that, like other diseases that involve an enzymatic dysfunction, the techniques needed for the diagnosis require a special management that makes it difficult to include in population studies.

In our hospital, we have started the genetic screening of glucogenosis and mucopolysacaridosis in DBS samples by massive sequencing in children with a dismorphology, hepatomegaly, or splecnomegaly not explained. Our index patient was diagnosed first by enzymatic deficient activity and genetically confirmed. However, the genetic screening of rare diseases with the possibility of treatment should be studied in newborns. We are trying to contribute to this goal with a genetic screening approach.

## Author contributions

**Conceptualization:** Myriam Ley-Martos, Juan M Guerrero, Pedro Paul-Sánchez, Hada C Macher.

**Data curation:** Juan M Guerrero, Hada C Macher.

**Formal analysis:** Hada C Macher.

**Funding acquisition:** Juan M Guerrero.

**Investigation:** Myriam Ley-Martos, Juan M Guerrero, Marta Lucas-Javato, Cristina Remon-García, Raul Garcia-Lozano, Cristobal Colón, Pablo Crujeiras, Daniel Rodrigues, Pedro Paul-Sánchez, Hada C Macher.

**Methodology:** Myriam Ley-Martos, Marta Lucas-Javato, Raul Garcia-Lozano, Cristobal Colón, Pablo Crujeiras, Daniel Rodrigues, Hada C Macher.

**Supervision:** Cristina Remon-García, Hada C Macher.

**Validation:** Myriam Ley-Martos, Raul Garcia-Lozano.

**Writing – original draft:** Myriam Ley-Martos, Juan M Guerrero, Hada C Macher.

**Writing – review & editing:** Juan M Guerrero, Hada C Macher.
